# Multiscale Design Principles for Nanocomposite Hydrogel-Based Triboelectric Nanogenerators

**DOI:** 10.1021/acs.nanolett.6c02504

**Published:** 2026-07-24

**Authors:** James Sangmin Choo, Jinsoo Na, Siheon Lee, Juhyuk Park

**Affiliations:** † Department of Materials Science and Engineering, 26725 Seoul National University, Seoul 08826, Republic of Korea; ⊥ Research Institute of Advanced Materials (RIAM), 26725 Seoul National University, Seoul 08826, Republic of Korea; § Interdisciplinary Program in Bioengineering, 26725 Seoul National University, Seoul 08826, Republic of Korea; ∥ SOFT Foundry Institute, 26725 Seoul National University, Seoul 08826, Republic of Korea

**Keywords:** nanocomposite hydrogels, triboelectric nanogenerators, nanofiller engineering, self-powered wearable sensors

## Abstract

Hydrogel-based triboelectric nanogenerators (H-TENGs) are soft, self-powered platforms for wearable sensing, human–machine interfaces, and biomechanical energy harvesting. Hydrogels offer mechanical compliance, ionic conductivity, and skin conformability, but practical H-TENGs remain limited by insufficient mechanical robustness, limited electrical or ionic transport, and limited output performance. Nanofiller engineering provides a materials-level route to address these limitations by reinforcing hydrogel networks, improving transport pathways, and tailoring interfacial charge behavior. This Mini-Review summarizes recent progress in nanocomposite H-TENGs with emphasis on mechanical reinforcement, electrical pathway engineering, nanofiller dispersion control, output modulation, and multifunctional integration. Rather than treating nanofillers as simple additives, we highlight how their dispersion, surface chemistry, and spatial arrangement govern hydrogel structure and device performance. Finally, we discuss challenges in matrix design, triboelectric interfaces, and device integration. These design principles may guide durable multifunctional nanocomposite hydrogel-based TENGs for wearable electronics under repeated deformation and long-term on-skin operation.

## Graphical abstract

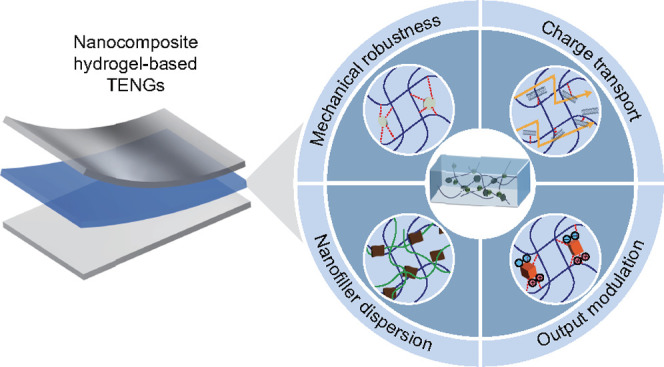

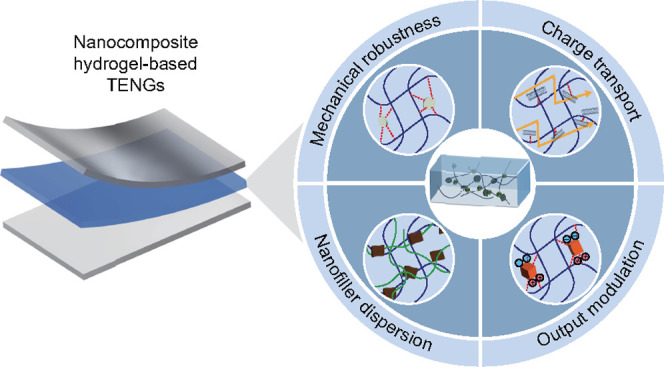

The rapid development of AI and the proliferation of data-intensive electronics have continuously increased the energy burden of modern electronic systems.^1^ At the same time, growing demand for wearable devices, portable devices, and distributed sensor platforms has further highlighted the importance of localized energy harvesting technologies that can reduce reliance on external power sources.^2,3^ In particular, within the field of wearable electronics, energy harvesting strategies capable of converting low-frequency biomechanical motions into usable electrical energy have attracted increasing attention.^4,5^

Among the various energy harvesting technologies investigated to date, triboelectric nanogenerators (TENGs) have received considerable interest because of their high output voltage, broad material selection, simple device architecture, and suitability for harvesting low-frequency mechanical energy.^6^ TENGs generate electrical output through the coupling of triboelectrification and electrostatic induction during repeated contact and separation between materials with different triboelectric polarities.^7^ Accordingly, material selection should be considered not only in terms of interfacial electronic states, surface chemistry, charge retention behavior, and processability but also with respect to mechanical and chemical stability.^8^ For these reasons, polymer-based materials with high flexibility and processability have been actively explored as triboelectric layers in TENG systems.^9^

More recently, with the increasing demand for wearable devices and on-skin electronics, extensive efforts have been devoted to incorporating hydrogels into TENGs because they are flexible, deformable, and capable of forming conformal contact with skin.^10–13^ Hydrogels can serve as soft conductive electrodes, triboelectric layers, or integrated functional matrices, and these characteristics are particularly advantageous for applications that require intimate contact and mechanical compliance, such as biosensing and human–machine interfaces. Nevertheless, practical hydrogel-based TENGs (H-TENGs) remain limited by insufficient mechanical robustness, limited electrical or ionic transport, and limited output performance.^14–16^

To overcome these limitations, increasing attention has been directed toward nanocomposite hydrogel-based TENGs, in which nanomaterials are introduced into hydrogel matrices.^17^ In polymeric materials, control over the microstructure is a critical factor in determining mechanical, transport, interfacial, and functional properties.^18–20^ In this context, nanofillers can alter the microscale pore architecture and polymer-network structure of hydrogels. These structural changes, together with the intrinsic properties of the fillers, can reinforce polymer networks, regulate electrical or ionic transport, tailor interfacial interactions, and impart additional multifunctionality.^21–23^ At the same time, however, nanofiller incorporation can also induce new trade-offs, including agglomeration, sedimentation, and nonuniform dispersion.^24^ As a result, recent research has moved beyond simple filler addition toward advanced nanofiller engineering strategies involving nanofiller dispersion control, surface/interface modification, controlled spatial arrangement, and multifunctional integration (Figure 1). From this perspective, this Mini-Review aims to elucidate the design principles that govern the structure and performance of nanocomposite hydrogel-based TENGs and to provide an outlook on realistic improvement pathways, thereby contributing to the development of H-TENGs for applications in soft wearable electronic devices.

**Figure 1:**
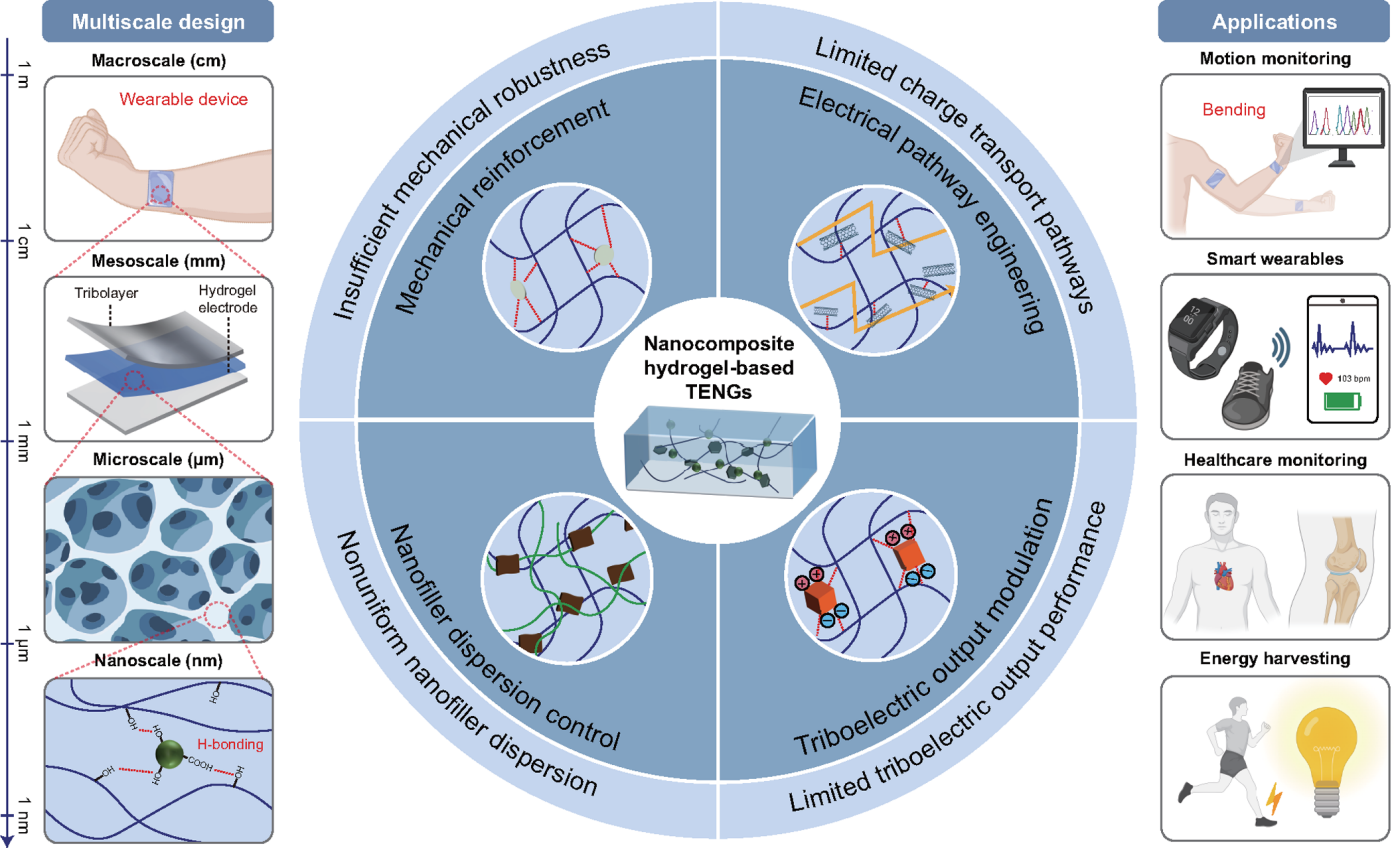
**Multiscale design framework of nanocomposite hydrogel-based TENGs.** Nanocomposite hydrogel-based TENGs still face key limitations, including insufficient mechanical robustness, limited charge transport pathways, nonuniform nanofiller dispersion, and limited triboelectric output performance. In the central circular diagram, the outer light-blue ring represents these challenges, while the inner dark-blue ring summarizes the corresponding design strategies for mechanical reinforcement, electrical pathway engineering, nanofiller dispersion control, and triboelectric output modulation. These strategies are organized from nanoscale filler–matrix interactions and microscale hydrogel structural engineering to mesoscale device assembly and macroscale wearable operation. These design principles support possible applications in motion monitoring, smart wearables, healthcare monitoring, and biomechanical energy harvesting.

## 1 Principles of Hydrogel-Based Triboelectric Nanogenerators (H-TENGs)

### 1.1 Triboelectric Nanogenerators (TENGs)

#### 1.1.1 Fundamentals of TENGs

Triboelectric nanogenerators (TENGs) harvest mechanical energy fundamentally through the synergistic interaction of triboelectrification and electrostatic induction.^25^ A typical TENG architecture consists of tribolayers, electrodes, and an external circuit. When two dissimilar materials come into physical contact, interfacial charge transfer occurs through contact electrification, and the extent and polarity of transferred charges are influenced by multiple factors, including the electronic structures of the paired materials, surface states, surface chemistry, contact conditions, and environmental factors^26^ (Figure 2A). In many polymer-based TENG systems, net charge transfer is commonly described in terms of electron transfer, leading to positive and negative surface charges on the paired triboelectric materials. Once the two charged surfaces are separated, the resulting interfacial charge imbalance generates a localized electric field that induces a potential difference between the electrodes. This potential gradient drives a directional electron flow through the external circuit to re-establish the electrostatic equilibrium of the system. As the separation distance changes periodically, the induced potential difference fluctuates, generating an alternating current (AC) output from the continuous mechanical motion.

**Figure 2:**
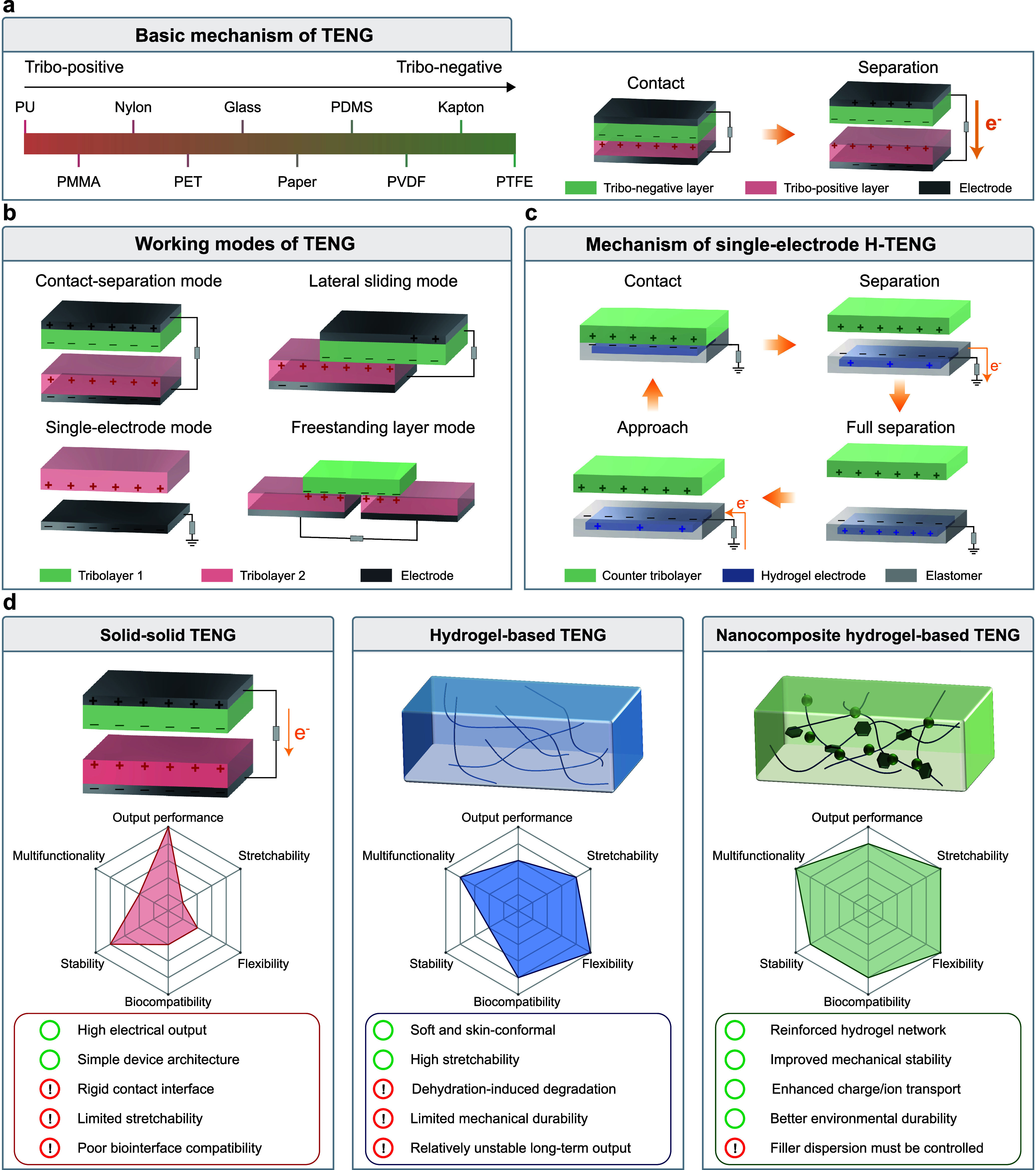
**Basic mechanism, working modes, and material comparison of TENG systems.** (a) Basic mechanism of TENGs, including the triboelectric series and contact–separation process. Contact between tribopositive and tribonegative materials generates opposite surface charges, and subsequent separation induces electron flow through the external circuit. (b) Four representative operation modes of TENGs, including vertical contact–separation, lateral sliding, single-electrode, and freestanding triboelectric-layer modes. (c) Contact–separation operation of a single-electrode H-TENG. Mechanical contact and separation generate triboelectric charges on the dielectric surface, inducing ion redistribution in the hydrogel and electron flow through the external circuit. (d) Comparison of solid–solid TENGs, hydrogel-based TENGs, and nanocomposite hydrogel-based TENGs. Nanocomposite hydrogel-based TENGs retain the softness and conformability of hydrogels while improving mechanical robustness, charge transport, output performance, and multifunctionality through nanofiller engineering.

#### 1.1.2 Operational Modes of TENGs

The operational architecture of TENGs is fundamentally categorized into four principal configurations: contact-separation mode, lateral sliding mode, single-electrode mode, and freestanding triboelectric-layer mode (Figure 2b). In the contact–separation mode, two distinct tribolayers repeatedly contact and separate in the normal direction, inducing a cyclic electron flow. The lateral sliding mode utilizes continuous in-plane relative displacement between charged surfaces, where changes in the overlapping area create a time-dependent potential difference. In the single-electrode mode, one electrode is connected to the external circuit and electrically referenced to the ground. Periodic approach and withdrawal of an external object alter the local electrostatic field, thereby inducing an electron flow through the circuit. Lastly, the freestanding triboelectric-layer mode employs a movable, charged dielectric layer sliding between two stationary electrodes without a permanent attachment. The freestanding triboelectric-layer mode is advantageous for harvesting mechanical energy from sliding or moving objects without directly wiring the moving triboelectric layer.

### 1.2 H-TENGs

Hydrogels, characterized by their 3D polymeric networks and high hydration capacity, can serve as either conformable tribolayers or flexible soft electrodes in TENG architectures.^19,27^ However, the intrinsic dehydration of water-rich networks remains a critical bottleneck, severely compromising the long-term operational and environmental stability of H-TENGs.^28^ To mitigate water evaporation, strategies such as incorporating humectants (e.g., glycerol), dissolving hygroscopic salts, or employing encapsulated and tubular device configurations have been extensively adopted to achieve a broad environmental adaptability.

Consequently, a prevalent H-TENG design utilizes conductive hydrogels as encapsulated flexible electrodes in single-electrode or contact–separation structures.^29^ In these systems, the hydrogel is embedded within an elastomeric matrix to ensure physical isolation from the external environment while preserving its hydration state. During mechanical stimulation, triboelectric charges generated on the encapsulating elastomer’s surface induce the rapid redistribution of mobile ions within the hydrogel (Figure 2c). This ion redistribution creates an excess-ion layer near the elastomer–hydrogel interface, while an electric double layer forms at the hydrogel–current collector interface, coupling ionic redistribution in the hydrogel with electron flow through the external circuit.

Despite these advantages, pristine H-TENGs remain limited by insufficient mechanical robustness, restricted electrical or ionic transport, and lower output performance than conventional solid–solid TENGs (Figure 2d). Incorporating functional nanofillers into hydrogel matrices provides a route to address these limitations by reinforcing polymer networks, regulating charge transport, modulating interfacial polarization, and introducing additional functions. These considerations motivate the development of nanocomposite hydrogel-based TENGs, in which filler–matrix interactions and network structures are used to improve device performance and expand wearable applications.

### 1.3 Output Performance of H-TENGs

The output performance of hydrogel-based triboelectric nanogenerators (H-TENGs) is governed by the coupled processes of interfacial charge generation, electrostatic induction, and electrode-mediated charge redistribution.^30^ During repeated contact and separation between the tribolayer and the counterface, surface charges are generated through contact electrification. The amount of triboelectric charge generated can be increased by enlarging the effective contact area, optimizing surface morphology, enhancing dielectric polarization, or incorporating polar or piezoelectric components near the triboelectric interface.^31^ These interfacial engineering strategies strongly influence the surface charge density and therefore affect the open-circuit voltage (*V*_OC_), transferred charge (*Q*_SC_), and short-circuit current (*I*_SC_). The measured output characteristics are further influenced by device geometry, dielectric properties, separation distance, capacitance variation, mechanical frequency or velocity, electrode polarization kinetics, internal resistance, and external circuit conditions.^32^ In particular, *I*_SC_ is closely related to the time-dependent charge-transfer process, whereas the output power depends strongly on load matching and impedance characteristics.

In most H-TENGs, the hydrogel electrode responds to the electrostatic field generated at the triboelectric interface through ion redistribution within the hydrated polymer network.^29^ Mobile ions migrate toward or away from the charged interface, forming electric-double-layer-like polarization, while electrons flow through the external metallic circuit to balance the induced potential difference. Therefore, the hydrogel electrode does not simply function as a passive current collector. Its ionic conductivity, electrical resistance, interfacial polarization behavior, and mechanical integrity collectively determine its electrical output. Added salts or ionizable components supply mobile ions, whereas the polymer network, water content, and microstructure regulate the effective mobility. In nanocomposite hydrogels, most fillers do not directly serve as mobile-ion sources but instead modify the local environment for ion migration by altering hydrated domains, pore structures, water retention, and interfacial interactions.

Conductive nanofillers and conducting polymers provide an additional route for improving electrode-mediated charge redistribution. Carbon-based nanofillers,^33^ MXenes,^34^ and PEDOT:PSS^35^ can form conductive or mixed conductive pathways within the hydrogel matrix, reducing internal resistance and facilitating charge collection at the hydrogel–current collector interface. Surface-functionalized fillers can also interact with water molecules and polymer chains through hydrophilic groups, hydrogen bonding, or interfacial microchannels, thereby influencing ion redistribution and interfacial polarization.^36^ However, output enhancement is generally maximized within an optimized filler-loading window. Insufficient filler content cannot form effective transport pathways, whereas excessive loading can induce aggregation, phase separation, and mechanical heterogeneity, which may increase resistance or disrupt charge transport.

Overall, high-performance H-TENGs require balanced control over triboelectric charge density, hydrogel electrode conductivity, ion redistribution kinetics, interfacial polarization, and mechanical resilience. Rather than simply maximizing filler loading, nanocomposite hydrogel design should optimize filler content, dispersion homogeneity, surface chemistry, and filler–polymer interactions to simultaneously improve charge transport, mechanical reliability, and output stability.

## 2 Nanofiller Engineering for Nanocomposite Hydrogel-Based Triboelectric Nanogenerators

To provide a comparative overview of nanofiller design, major nanofiller classes used in nanocomposite hydrogel-based TENGs are summarized in Table 1 according to their representative examples, primary roles, advantages, and limitations. This class-level comparison highlights general trends in nanofiller function and design trade-offs across different systems. Representative nanocomposite hydrogel-based TENGs are further summarized in Table S1, including hydrogel/nanofiller compositions, key nonoutput material parameters, and reported *V*_OC_ and *I*_SC_ values.

**Table 1:** Comparative Roles and Design Considerations of Major Nanofiller Classes in Nanocomposite Hydrogel-Based TENGs^a^

nanofiller class	representative examples	primary role	advantages	limitations
nanoclays	Laponite^37,38,41^	physical cross-linking nodes	improved toughness, stretchability, and mechanical durability under repeated deformation	limited direct contribution to triboelectric charge modulation due to electrically insulating nature
biopolymer nanofillers	nanochitin;^39^ CNF;^56–58^ TOCNF^59^	reinforcing networks; dispersion stabilizers	hydrogel-network stabilization and active-filler dispersion through hydrogen bonding and high-aspect-ratio structures	passive reinforcing or stabilizing role without conductive or polar fillers
MOFs	ZIF-8;^40^ UiO-66(Zr)-F_4_ ^60^	coordination nodes; porous or polar domains	tunable multipoint interactions, network reinforcement, charge trapping, and interfacial polarization	chemistry-dependent functionality and limited direct output modulation in nonpolar MOFs
carbon-based fillers	GO;^41,42,49^ CNT derivatives;^46,48,61,62^ GNP;^45,63^ activated carbon;^44^ carbon ink^47^	conductive or charge-trapping carbon networks	enhanced conductivity, charge redistribution, and interfacial polarization	aggregation-prone conductive networks requiring dispersion control
MXenes	Ti_3_C_2_T_*x*_ MXene;^50–54,56,57^ modified MXene^59,64^	conductive and polar 2D nanosheets	ion/electronic transport, edl modulation, interfacial polarization, and multifunctional hydrogel design	oxidation and nanosheet restacking in aqueous or ambient environments
oxide, piezoelectric, and ferroelectric fillers	SiO_2_@ZnO;^43^ BaTiO_3_;^65–67^ BaTiO_3_@gliadin^68^	rigid reinforcing or polarization-active domains	dielectric, piezoelectric, or polarization-assisted charge induction and output modulation	dispersion-, coating-, and localization-dependent output enhancement
semiconductor and quantum-dot fillers	SnS_2_/SnS nanoflakes;^69^ CQDs^70^	photoactive or antibacterial functional domains	light-responsive output modulation, antibacterial activity, and multifunctional sensing capability	application-specific functionality with limited general reinforcement or conductive-network contribution
liquid metal nanofillers	Ga-based liquid-metal nanospheres^71^	deformable conductive networks	soft, stretchable, and highly conductive charge-collection pathways	precipitation, phase separation, leakage, or crack formation without interfacial stabilization

a Abbreviations: CNF, cellulose nanofibril; TOCNF, TEMPO-oxidized cellulose nanofibril; MOF, metal–organic framework; ZIF-8, zeolitic imidazolate framework-8; GO, graphene oxide; CNT, carbon nanotube; GNP, graphene nanoplatelet; EDL, electric double layer; CQD, carbon quantum dot; LM, liquid metal; DL@LM, demethylated-lignin-coated liquid metal.

### 2.1 Mechanical Reinforcement

The mechanical performance of H-TENGs is one of the key criteria for wearable applications, because the hydrogel components must withstand repeated tensile and compressive stresses during long-term use. Although hydrogels generally exhibit high flexibility, many hydrogel matrices still suffer from insufficient strength or limited stretchability, depending on their polymer network structure (Figure 3a). To address these limitations, nanofillers such as inorganic clay nanoparticles,^37,38^ nanochitin,^39^ metal–organic frameworks (MOFs),^40^ graphene oxide (GO),^41,42^ and oxide ceramic nanoparticles^43^ have been incorporated into hydrogel matrices. These nanofillers can reinforce the hydrogel network by increasing the effective cross-linking density and introducing additional energy dissipation pathways through physical and chemical cross-linking. Nanofillers are particularly useful for mechanical reinforcement because their high specific surface area and surface functional groups enable strong filler–matrix interactions, including hydrogen bonding, electrostatic interactions, and metal ion coordination.

**Figure 3:**
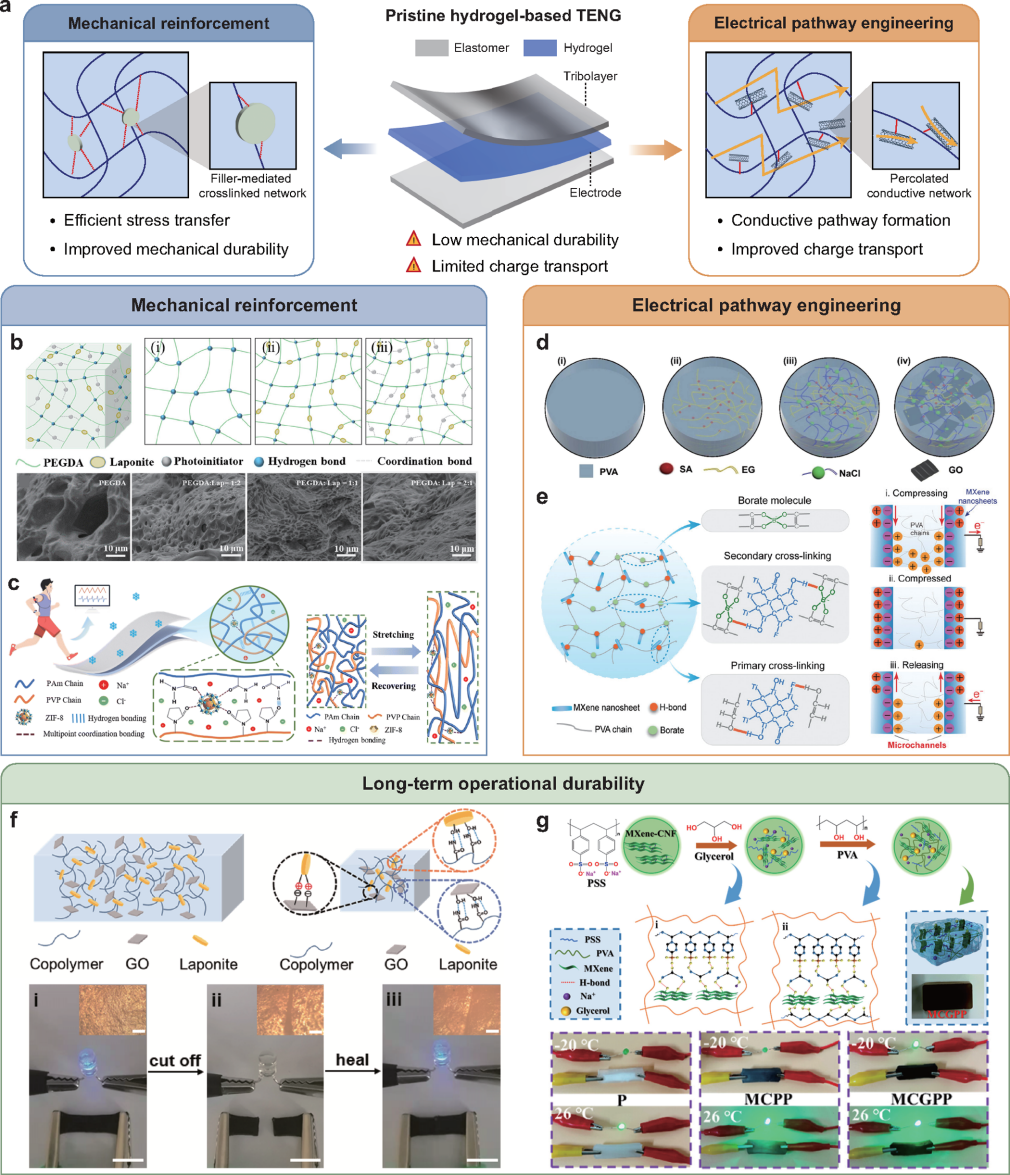
**Nanofiller engineering for mechanical reinforcement and electrical pathway engineering in nanocomposite hydrogel-based TENGs.** (a) Schematic illustration of pristine hydrogel-based TENGs with insufficient mechanical robustness and limited electrical or ionic transport and nanofiller-enabled strategies for hydrogel-network reinforcement and conductive-pathway formation. (b, c) Filler-mediated physical network reinforcement in nanocomposite hydrogels. (b) Laponite-reinforced PEGDA hydrogel network. Reproduced from ref 37. Copyright 2023 American Chemical Society. (c) ZIF-8-reinforced PAm/PVP double-network hydrogel. Adapted with permission from ref 40. Copyright 2025 the authors of ref 40, exclusive license to the Royal Society of Chemistry. (d, e) Electrical pathway engineering in conductive hydrogel electrodes. (d) GO-incorporated PVA/SA hydrogel electrode. Reproduced with permission from ref 49. Copyright 2025 Elsevier. (e) MXene/PVA hydrogel electrode for charge transport and streaming-vibration-potential-assisted output enhancement. Adapted with permission from ref 52. Copyright 2021 Wiley-VCH. (f, g) Long-term operational durability of hydrogel-based TENGs. (f) GO/Laponite ionic hydrogel with reversible filler–matrix and filler–filler interactions for self-healing and output recovery. Adapted from ref 41. CC BY-NC 3.0. (g) MXene–CNF/PSS/glycerol/PVA hydrogel with ion-conducting microchannels and water-retentive/antifreezing design for temperature-tolerant operation. Adapted from ref 56. Copyright 2024 American Chemical Society.

Among them, Laponite is a disk-shaped synthetic nanoclay that can act as a multifunctional physical cross-linker within hydrogel networks. When dispersed in a polymer matrix, Laponite can interact with polymer chains through reversible secondary interactions, while its rigid nanosheets serve as energy dissipation sites during deformation. Therefore, Laponite incorporation can improve stretchability, toughness, and resistance to mechanical failure without sacrificing the soft nature of the hydrogel. Li et al.^37^ incorporated Laponite into a poly­(ethylene glycol) diacrylate (PEGDA) hydrogel matrix to construct additional physical networks through polymer–filler interactions. They optimized the PEGDA/Laponite ratio and showed that Laponite promoted the formation of a more interconnected porous hydrogel microstructure (Figure 3b). In this system, secondary interactions between PEGDA chains and Laponite nanoparticles reinforced the chemically cross-linked PEGDA network. This filler-mediated network improved the flexibility, tensile resistance, pressure tolerance, and long-term durability of the biodegradable H-TENG. Similarly, Zeng et al.^38^ introduced Laponite into an OEG copolymer/poly­(3,4-ethylenedioxythiophene):poly­(styrenesulfonate) (PEDOT:PSS) hydrogel system. Laponite interacted with the OEG polymer chains and improved the dispersion and stability of PEDOT:PSS through interactions with PEDOT:PSS colloidal particles. The resulting multiphysically cross-linked network enhanced stretchability, self-healing, tissue adhesion, and mechanical stability.

Metal–organic frameworks (MOFs) can reinforce hydrogel matrices through their large surface areas and abundant coordination sites. Xu et al.^40^ incorporated zeolitic imidazolate framework-8 (ZIF-8) nanoparticles into a polyacrylamide/poly­(vinylpyrrolidone) (PAm/PVP) double-network hydrogel, where the particles formed multipoint interactions with polymer chains, including metal coordination, hydrogen bonding, and electrostatic interactions (Figure 3c). These interactions connected the PAm/PVP network more effectively and acted as dynamic physical cross-linking points during deformation. The ZIF-8-containing hydrogel showed a more compact and integrated porous structure, which helped to dissipate stress while maintaining elastic recovery. As a result, the hydrogel exhibited improved stretchability, low mechanical hysteresis, compression recovery, and high cyclic stability.

### 2.2 Electrical Pathway Engineering

Electrical enhancement in nanocomposite hydrogel-based TENGs is commonly achieved by incorporating conductive nanofillers into hydrogel electrodes, mainly carbon-based nanofillers^44–49^ and MXene nanosheets^50–54^ (Figure 3a). In this section, electrical pathway engineering primarily refers to the formation of conductive or mixed conductive pathways within the hydrogel electrode. Once sufficiently connected, these pathways reduce the internal resistance of the hydrogel electrode, facilitate charge collection at the hydrogel–current collector interface, and improve electrode-mediated charge redistribution during repeated contact–separation cycles.^55^ However, the enhancement is not simply proportional to filler loading. Insufficient filler content cannot establish continuous conductive pathways, whereas excessive nanofiller content often causes aggregation, phase separation, increased local resistance, or disruption of the polymer network.^36^ These defects can hinder charge transport and reduce output performance. Therefore, electrical performance is generally maximized at an optimized filler concentration that balances conductive network formation, filler dispersion, and hydrogel network integrity.

Manchi et al.^49^ incorporated GO sheets into a poly­(vinyl alcohol)/sodium alginate (PVA/SA) hydrogel and further introduced sodium chloride (NaCl) as an ionic conductor (Figure 3d). In this system, PVA and SA formed a flexible hydrogen-bonded network, while Na^+^ and Cl^–^ ions provided mobile charge carriers. GO sheets further enhanced the hydrogel conductivity by forming additional charge-transport pathways. The TENG output increased with GO loading and reached the optimum at 0.75 wt % GO, giving an output voltage of approximately 495 V, current of 22 μA, and charge density of 125 μC·m^–2^. At higher GO loading, the output decreased to approximately 400 V and 14 μA, indicating that excessive GO disrupted dispersion and network continuity. The optimized PVA/SA@GO hydrogel also increased the power density from 1.4 W·m^–2^ for PVA/SA to 4.2 W·m^–2^, showing that GO-based enhancement is strongly controlled by filler dispersion and conductive network formation.

Zhang et al.^46^ used hydroxylated multiwalled carbon nanotubes (HO-mCNT), PVA, and tannic acid (TA) to construct a dynamic conductive hydrogel network. HO-mCNT formed conductive pathways in the PVA matrix, while TA and hydroxylated nanotube surfaces generated hydrogen-bonding interactions with PVA chains. This structure increased the conductivity from 0.79 mS m^−1^ for the pristine hydrogel to 1.94 mS·m^–1^ for the optimized HO-mCNT hydrogel. The corresponding TENG output voltage increased from 255 to 398 V after optimized HO-mCNT incorporation. However, further HO-mCNT addition reduced the output to 312 V, because excessive CNT loading caused aggregation and disrupted the uniform conductive network. This result indicates that CNT-based electrical enhancement requires balanced filler loading and continuous charge-transport pathways.

MXene nanosheets enhance hydrogel electrodes by combining interfacial cross-linking with ion-transport modulation. Luo et al.^52^ introduced MXene into a PVA hydrogel, where MXene surface functional groups formed hydrogen bonds with PVA chains and promoted primary and secondary cross-linking (Figure 3e). The MXene-associated microchannels also enabled water and ion migration during compression and release, generating an additional streaming vibration potential. The optimized MXene/PVA hydrogel increased the output voltage from 60 V for undoped PVA to 230 V, while the current increased from 60 nA to 270 nA. In another MXene-based design, Zhang et al.^54^ introduced MXene into a polyacrylamide/sodium alginate/tannic acid (PAM/SA/TA) hydrogel, where PAM/SA formed the hydrogel backbone, TA provided dynamic hydrogen bonding, and MXene established conductive pathways in the self-healable network. The output increased with MXene content up to 2%, reaching approximately 150 V and 2 μA, but decreased at 3% because MXene aggregation hindered ion transport and increased hydrogel resistance. After repeated damage-healing cycles, the device maintained stable electrical output, indicating that the dynamic hydrogel network could reconstruct conductive pathways after mechanical damage. These studies show that MXene improves H-TENG performance through conductive pathway formation, microchannel-assisted charge transport, and network recoverability.

### 2.3 Long-Term Operational Durability

Although H-TENGs are soft and conformable, their output can become unstable during repeated deformation, mechanical damage, dehydration, or temperature variation.^28^ These issues are important for wearable operation because the hydrogel electrode must retain both structural integrity and charge transport capability during continuous use. Long-term operational durability is therefore determined by the combined stability of the hydrogel network, conductive pathways, triboelectric interfaces, and device architecture. In nanocomposite hydrogel-based TENGs, functional nanofillers can contribute to durability by reinforcing the hydrogel network, introducing reversible interactions, suppressing crack propagation, and maintaining conductive or ion transport pathways. Together with these filler-mediated effects, water-retentive solvent systems, mobile-ion sources, and encapsulated architectures can further reduce dehydration, suppress freezing, maintain ionic conductivity, and limit interfacial failure.

#### 2.3.1 Damage Recovery and Cyclic Output Stability

Mechanical damage in H-TENGs can disrupt the hydrogel network and conductive pathways, resulting in reduced or unstable electrical output. Therefore, damage recovery is an important requirement for long-term operation. Self-healing strategies have been introduced to restore mechanical integrity and electrical continuity after damage. Li et al.^41^ developed an ultrastretchable and healable TENG using a Laponite/poly­(AMPS-*co*-AA-*co*-DMAPMA)/GO–LiCl ionic hydrogel electrode (Figure 3f). In this hydrogel, GO and Laponite acted as collaborative physical cross-linking points by forming dynamic hydrogen bonds with the amide groups of the polymer chains, while reversible electrostatic interactions between GO and Laponite further supported network recovery. These reversible filler–matrix and filler–filler interactions enabled the damaged hydrogel to recover ion-conduction pathways after mechanical damage. After being cut and healed at 80 °C, the hydrogel recovered 99% of its fracture strain after 36 h, and the corresponding hydrogel-based TENG recovered its electrical output after healing. The device also maintained stable output over more than 3000 contact–separation cycles. This result shows that nanofillers can contribute to damage recovery by forming reversible interactions that restore both hydrogel integrity and charge transport.

#### 2.3.2 Water-Retentive and Temperature-Tolerant Operation

Environmental durability is also required for H-TENGs because water evaporation and freezing directly affect hydrogel mechanics, ionic conductivity, and output stability. Water-retentive solvent systems and mobile-ion sources are commonly used to reduce water loss and maintain ion transport under variable temperature conditions. Liu et al.^56^ reported an ion-conducting PVA nanocomposite hydrogel composed of MXene–CNF microchannels, PSS, glycerol, and PVA. In this hydrogel, MXene–CNF microchannels provided pathways for directional ion transport, CNF helped immobilize MXene nanosheets, and PSS introduced mobile Na^+^ counterions (Figure 3g). Glycerol was introduced as a water-retentive and antifreezing component through hydrogen-bonding interactions with water and the PVA matrix. As a result, the hydrogel retained flexibility at −20 °C and showed high moisture retention after storage at 26 and 60 °C. The corresponding hydrogel-based TENG generated open-circuit voltages of 352, 368, and 362 V at −20, 26, and 60 °C, respectively. This example shows that temperature-tolerant H-TENG operation can be achieved by combining nanofiller-mediated ion-transport pathways with matrix-level water-retention and antifreezing design.

Overall, long-term operational durability in nanocomposite hydrogel-based TENGs should be evaluated not only by initial mechanical properties, conductivity, or output values, but also by output retention after damage, repeated contact–separation cycles, mechanical deformation, storage, and temperature variation. These studies indicate that durable H-TENGs require integrated matrix, filler, and device-level design to maintain hydrogel integrity, charge transport, and interfacial stability under practical operating conditions.

## 3 Advanced Nanofiller Engineering

The mechanical reinforcement and electrical pathway engineering discussed in section 3 establish the basic roles of nanofillers in nanocomposite hydrogel-based TENGs. Building on these strategies, advanced nanofiller engineering extends beyond simple filler addition by controlling filler dispersion, surface chemistry, spatial distribution, orientation, and functional activity within hydrogel matrices. These strategies allow nanofillers to act not only as reinforcing or conductive additives but also as interfacial stabilizers, dynamic cross-linking nodes, charge-modulating components, piezoelectric domains, and multifunctional active elements. In this section, advanced nanofiller engineering is discussed in terms of nanofiller dispersion control, output modulation, and multifunctional integration (Figure 4A).

**Figure 4:**
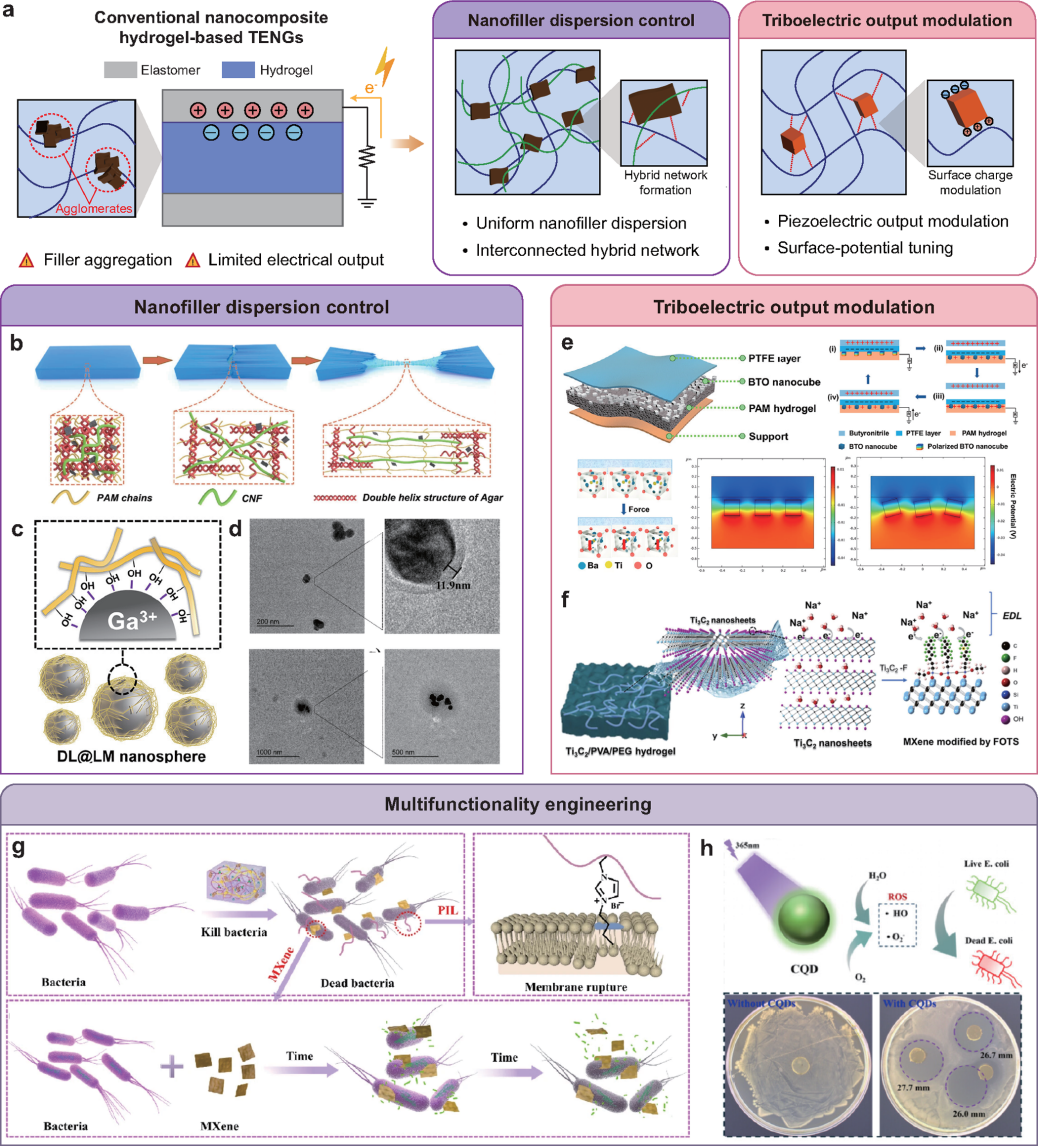
**Advanced nanofiller engineering and triboelectric output modulation in nanocomposite hydrogel-based TENGs.** (a) Schematic illustrations of nanofiller aggregation and limited electrical output in conventional nanocomposite H-TENGs and advanced nanofiller engineering strategies for nanofiller dispersion control and output modulation. (b–d) Dispersion-control strategies based on synergistic filler incorporation, surface modification, and encapsulation-like structural stabilization. (b) Multinetwork-structured PAM-AG/CNF-MXene triboelectric hydrogel. Reproduced with permission from ref 57. Copyright 2024 Elsevier. (c) Demethylated lignin-coated liquid-metal nanospheres for conductive hydrogel formation. Reproduced from ref 71. Copyright 2025 American Chemical Society. (d) BaTiO_3_@gliadin microcapsule-reinforced hydrogel network. Adapted with permission from ref 68. Copyright 2026 Elsevier. (e, f) Representative triboelectric output modulation strategies. (e) Unsymmetrical BaTiO_3_ distribution for piezoelectricity-assisted output enhancement. Adapted from ref 65. Copyright 2022 American Chemical Society. (f) Silane-modified MXene/PVA hydrogel for enhanced streaming vibration potential. Reproduced with permission from ref 64. Copyright 2024 Elsevier. (g, h) Multifunctionality engineering using functional nanofillers in hydrogel-based TENGs. (g) MXene-containing ion-conducting double-network composite hydrogel with antibacterial activity and environmental tolerance. Adapted from ref 75. CC BY 4.0. (h) CQD-containing stretchable antibacterial hydrogel electrode for biomechanical sensing. Adapted from ref 70. Copyright 2024 American Chemical Society.

### 3.1 Nanofiller Dispersion Control

A major bottleneck in nanofiller engineering for nanocomposite hydrogel-based TENGs is the nonuniform dispersion of multifunctional nanofillers within highly hydrated polymer networks. Although conductive or polar nanofillers can improve mechanical strength, charge transport, interfacial polarization, and triboelectric output, excessive filler loading often induces aggregation because of strong filler–filler interactions. This aggregation disrupts the continuous polymer network, decreases effective interfacial contact between the filler and hydrogel matrix, and ultimately compromises both mechanical durability and electrical output. Therefore, recent studies have focused not only on selecting high-performance nanofillers, but also on improving their dispersion stability through synergistic filler incorporation,^56,57,59,63,72^ surface modification,^61,73^ and encapsulation-like structural stabilization.^68,71^

#### 3.1.1 Synergistic Filler Incorporation

MXene has been widely introduced into conductive hydrogels because of its metallic conductivity, hydrophilic surface groups, and large specific surface area. However, MXene nanosheets are prone to oxidation and restacking in aqueous and oxidative environments, especially during hydrogel polymerization. To address this limitation, Chen et al.^59^ proposed a bioinspired 2,2,6,6-tetramethylpiperidin-1-oxyl (TEMPO)-oxidized cellulose nanofiber/tannic acid-coated MXene (TOCNF/TA@MXene) system, in which tannic acid (TA) encapsulated the MXene surface through catechol-rich interactions, while TOCNF acted as a leaf-vein-like support to separate and stabilize MXene nanosheets. The resulting TOCNF/TA@MXene dispersion remained stable without obvious aggregation for 11 days under H_2_O_2_ exposure, whereas pristine MXene aggregated within 3 days. This improved dispersion promoted a uniform 3D hydrogel network, leading to high stretchability of over 800%, reliable fatigue resistance, and a TENG output of 106 V, approximately 2 μA, and 31 nC in single-electrode mode. Similarly, Wei et al.^57^ used CNF as a sacrificial reinforcing component and MXene-dispersion scaffold in a PAM–agar hydrogel. Hydrogen-bonding and Coulombic interactions shifted the zeta potential of MXene from −17.6 to −33.9 mV, stabilizing the dispersion for 3 months (Figure 4b). This multibranched network yielded a highly stretchable and conductive triboelectric hydrogel with an elongation at break of 2303%, tensile strength of 0.54 MPa, conductivity of 161.44 mS·m^–1^, and a PACM-TENG output of 220 V and 5.93 μA.

A similar dispersion-oriented strategy was used by Luu et al.^63^ for graphene-based hydrogel electrodes. In this work, graphene nanoplatelets (GNPs) were first dispersed with poly­(4-styrenesulfonic acid) (PSS), where hydrophobic and π–π interactions helped stabilize GNPs, while hydrophilic sulfonic acid groups prevented graphene precipitation. After incorporation into a poly­(vinyl alcohol)/poly­(ethylene oxide) (PVA/PEO) hydrogel network, GNP-PSS formed hydrogen bonding with PVA and enabled uniform elemental distribution throughout the hydrogel. The GNP concentration was used to control the hydrogel conductivity and TENG output, with 20% GNP-PSS giving the highest output. However, further GNP-PSS loading reduced performance because excessive accumulation inhibited charge and ion transport. This result highlights that nanofiller dispersion control is not simply a processing detail, but a direct determinant of the electrical output window in nanocomposite hydrogel-based TENGs.

#### 3.1.2 Surface Modification

Surface modification provides another effective route to suppress nanofiller aggregation and improve interfacial compatibility with the hydrogel matrix. Li et al.^73^ modified carbon nanotubes with polydopamine (PDA) before incorporating them into a PAM hydrogel. The PDA layer enhanced the compatibility between carbon nanotubes (CNTs) and the PAM matrix and promoted CNT dispersion through catechol-mediated interactions. At the same time, PDA introduced abundant hydrogen bonding and π–π stacking sites, which endowed the PDA@CNT/PAM hydrogel with self-healing, self-adhesive, and conductive properties. The resulting hydrogel exhibited a self-healing efficiency of 97.3%, a gauge factor of 3.93 at 400% strain, and a fast response time of 76 ms, while the PDA@CNT/PAM-based triboelectric pressure sensor showed self-powered pressure detection without an additional power supply.

#### 3.1.3 Encapsulation-like Structural Stabilization

Du et al.^71^ further demonstrated a coating-based stabilization strategy using demethylated lignin-coated liquid metal nanospheres (DL@LM) (Figure 4c). Liquid metal is attractive as a soft conductive filler, but its high density, high surface tension, and poor aqueous stability can cause precipitation, phase separation, and crack formation in the hydrogel matrix. In this system, demethylated lignin served as an interfacial stabilizer for liquid metal nanospheres. Its phenolic hydroxyl and catechol groups interacted with the oxide layer of liquid metal through hydrogen bonding and Ga^3+^ coordination, thereby suppressing aggregation and improving compatibility with PAA chains. Compared with pristine liquid metal particles, DL@LM nanospheres showed a smaller and more uniform particle size distribution, and the resulting hydrogel achieved high toughness of 3.77 MJ·m^–3^, conductivity of 2.14 mS·cm^–1^, strong adhesion, and self-healing behavior. When applied as a single-electrode TENG, the hydrogel generated a high open-circuit voltage of 272 V and maintained stable output over 1000 cycles. Similarly, Zong et al.^68^ used polydopamine-modified barium titanate@gliadin (PBT@Gli) microcapsules as a biopolymer-assisted encapsulation platform, where the gliadin shell improved BT dispersion and interfacial compatibility with the PAM matrix while creating hierarchical charge-trapping interfaces. The optimized PGPBT hydrogel containing 40% PBT@Gli delivered a TENG output of 300 V, 12.6 μA, and 1.5 W·m^–2^, representing a 45.8% increase in open-circuit voltage compared with the physically blended PAM/Gli/PBT control, and maintained stable output over 3000 cycles.

Overall, these studies show that uniform nanofiller distribution is a prerequisite for reliable performance of nanocomposite hydrogel-based TENGs. Synergistic filler systems such as TOCNF/TA@MXene and GNP-PSS improve dispersion by combining structural support with interfacial stabilization, whereas surface-modified or coated fillers such as PDA@CNT and DL@LM enhance matrix compatibility through hydrogen bonding, π–π interactions, and metal–ligand coordination. By suppressing aggregation and maintaining continuous mechanical and conductive networks, these strategies enable nanofillers to contribute effectively to mechanical robustness, charge transport, self-healing, adhesion, and stable triboelectric output.

### 3.2 Output Modulation

In addition to reducing internal resistance through conductive pathway formation, as discussed in section 3.2, advanced nanofiller engineering can further modulate the electrical output of H-TENGs by regulating charge generation and interfacial polarization. Unlike electrical pathway engineering, which mainly improves electrode-mediated charge collection, output modulation focuses on controlling the spatial arrangement, surface chemistry, and polarization behavior of nanofillers within the hydrogel-based devices. Representative approaches include engineered unsymmetrical designs,^65,74^ filler alignment,^58^ surface chemical modification,^64^ and piezoelectric nanofiller incorporation.^60,66,67^ These strategies provide additional routes to enhance the H-TENG output beyond simple conductivity enhancement.

#### 3.2.1 Engineered Unsymmetrical Design

The spatial positioning of active fillers relative to the triboelectric interface is a key design parameter for output modulation. Wang et al.^65^ demonstrated this principle using an unsymmetrical PAM/BTO composite hydrogel in which BTO nanocubes were concentrated on one side of the hydrogel, facing the PTFE triboelectric layer (Figure 4e). Under mechanical stress, the BTO nanocubes generated piezoelectric charges near the contact interface, thereby increasing electrostatic induction and strengthening the TENG output. The optimized H-BTO-2-based TENG generated 52.92 V, 0.95 μA, and 17.27 nC, and its open-circuit voltage was approximately three times higher than that of a symmetrically dispersed BTO hydrogel device. The device also exhibited a sensitivity of 7.91 V·N^–1^ under forces below 1 N, with a response time of 70 ms and a decay time of 52 ms. This result demonstrates that output enhancement depends not only on the type and amount of piezoelectric filler, but also on its position relative to the triboelectric interface.

#### 3.2.2 Filler Alignment

In addition to asymmetric filler localization, the directional alignment of conductive fillers can modulate the output by controlling the anisotropic arrangement of conductive or polar components within the hydrogel electrode. Zeng et al.^58^ fabricated a PAM/CNF/MXene conductive hydrogel using electrohydrodynamic (EHD) printing assisted by in situ photopolymerization. During EHD printing, MXene nanosheets in the hydrogel precursor were directionally arranged under an electrostatic field, forming stable conductive channels within the PAM/CNF hydrogel network. This ordered MXene arrangement improved the conductivity of the hydrogel by 58% compared to manual injection, while CNF and MXene formed hydrogen-bonded interpenetrating networks with PAM. As a result, the PCM hydrogel showed a tensile strain of 550%, a gauge factor of 6.73, and a response/recovery time of 100/110 ms, and the PCM-TENG generated an open-circuit voltage of 67.5 V at 100% strain. These findings suggest that filler alignment can modulate the TENG output by constructing ordered conductive pathways within the hydrogel electrode.

#### 3.2.3 Surface Chemical Modification

Surface chemical modification offers another strategy for regulating interfacial charge behavior. Lai et al.^64^ modulated the TENG output by tuning the surface chemistry of MXene nanosheets. In their PVA/PEG hydrogel system, Ti_3_C_2_ MXene nanosheets were introduced to generate streaming vibration potential (SVP), and the MXene surfaces were further modified with 3-chloropropyltrimethoxysilane (CPTMS) or 1*H*,1*H*,2*H*,2*H*-perfluorooctyltriethoxysilane (FOTS) (Figure 4f). These silane-modified MXene nanosheets increased the charge density in the electric double layer formed at the MXene–water interface, thereby strengthening the SVP contribution. The output followed the order F-MPPh > Cl-MPPh > MPPh > PPh TENG, showing that fluorinated MXene provided the strongest output modulation. The Cl-MPPh and F-MPPh TENGs achieved open-circuit voltages of 190 and 212 V, respectively, and the F-MPPh device showed clear voltage responses under stretching, twisting, wrist-bending, and elbow-bending motions.

#### 3.2.4 Piezoelectric Nanofiller Incorporation

Piezoelectric nanofiller incorporation provides a route to enhance charge generation in hydrogel electrodes through filler-induced polarization and ion-transport modulation. Sarkar et al.^60^ incorporated UiO-66­(Zr)-F_4_ MOF into a PVA/polypyrrole (PPy) hydrogel to obtain a UiO-66­(Zr)-F_4_ MOF-reinforced piezoelectric hydrogel (UFMPH). The fluorinated MOF introduced piezoelectric polarization and enhanced ion transport, leading to a tensile stress of 6.88 MPa and conductivity of 1.96 S·m^–1^. By combining this piezoelectric hydrogel electrode with a leaf-inspired microstructured PDMS charge-generating layer, the resulting SUHTENG achieved a maximum power density of 0.78 W·m^–2^ and a sensitivity of 29.3 V·kPa^–1^. This study shows that piezoelectric fillers can increase charge generation and improve self-powered sensing performance in hydrogel-based TENGs.

Overall, these studies demonstrate that the H-TENG output can be modulated through the spatial distribution, directional alignment, surface chemistry, and piezoelectric response of nanofillers. Advanced nanofiller engineering therefore provides an active design framework for improving the nanocomposite H-TENG output beyond simple conductivity enhancement.

### 3.3 Multifunctionality Engineering

Beyond electrical output enhancement, nanofiller incorporation has also been used to impart optical responsiveness,^69^ near-infrared (NIR) photothermal functionality,^62^ and antibacterial activity^70,75^ to H-TENGs.

As a representative example of optically responsive nanofiller design, Das et al.^69^ introduced photoactive SnS_2_/SnS nanoflakes into a 3D-printed PVA hydrogel to fabricate a photoinduced triboelectric nanogenerator. In this system, the SnS_2_/SnS nanoflakes served as light-responsive semiconductor fillers within the hydrogel tribopositive layer. Under light illumination, photogenerated electron–hole pairs enhanced charge trapping through a mutual coupling effect, increasing the open-circuit voltage from 29.0 to 37.5 V and the short-circuit current from 1.23 to 1.58 μA. The device also achieved a power density of 5.4 μW·cm^–2^ under illumination, demonstrating that photoactive nanofillers can convert H-TENGs into light-modulated energy-harvesting platforms.

This optical functionality can be further extended from light-modulated output enhancement to photothermal bioapplications. Yang et al.^62^ developed a self-healing and NIR-responsive multifunctional TENG by incorporating PDA-modified CNTs into a PVA hydrogel electrode sandwiched between self-healing silicone elastomer layers. The PDA-CNTs improved electrical conductivity, enhanced filler dispersion, and provided NIR photothermal conversion capability. Under 808 nm laser irradiation, the TENG temperature increased gradually from 25 to about 60 °C within 5 min, enabling photothermal treatment to assist joint-motion recovery. In addition, the device maintained self-healing behavior, with the original structure and electrical output recovering within 10 min after mechanical damage. This work shows that conductive nanofillers can simultaneously provide self-powered sensing, self-healing reliability, and photothermal therapeutic functionality.

Beyond optical and photothermal responses, nanofiller-containing hydrogels have also been designed to combine environmental tolerance with biological functionality. Zhao et al.^75^ reported a poly­(ionic liquid)/MXene/PVA double-network ion-conducting hydrogel (PMP DN ICH) with broad environmental tolerance and multifunctional device integration (Figure 4g). MXene nanosheets acted as physical cross-linkers through hydrogen bonding with the polymer network, while the poly­(ionic liquid) component improved ionic conductivity and temperature resistance. The resulting PMP DN ICH exhibited high ionic conductivity of 63.89 mS·cm^–1^ at 25 °C, temperature resistance from −60 to 80 °C, long-term stability, high oxidation resistance, antibacterial activity, and adhesion. The antibacterial behavior originated from both imidazolium-containing poly­(ionic liquid) chains and MXene nanosheets, giving inhibition zones of 24 mm against both *E. coli* and *S. aureus* and antibacterial rates of 99.91% and 99.98%, respectively. When used as the electrode/current collector in a single-electrode TENG, the PMP DN ICH-based device delivered 66.0 V, 0.18 μA, 20.6 nC, and a maximum power density of 77.3 mW·m^–2^, while also being integrated into wireless strain sensors, thermal sensors, and all-solid-state supercapacitors.

A related antibacterial strategy was demonstrated by Song et al.,^70^ who developed a stretchable antibacterial hydrogel electrode for wearable TENGs using a CQDs/LiCl/agar-PAAm double-network hydrogel (Figure 4h). In this system, agar regulated the pore size, pore spacing, and cross-linking density of the PAAm-based hydrogel network, thereby improving ion migration, mechanical strength, and electrical conductivity. With 3 wt % agar, the hydrogel electrode showed a stretchability of 349% and conductivity of 1.87 S·m^–1^, while the tensile strength and conductivity increased by 211% and 719%, respectively, compared with the hydrogel without agar. The corresponding single-electrode TENG generated 120 V, 5.7 μA, 25 nC, and a maximum power density of 0.9 W·m^–2^, with stable output over 6000 cycles. Importantly, the CQDs served as the antibacterial nanofiller rather than the primary mechanical or conductive reinforcing component, producing an inhibition zone of 26.8 mm against *E. coli* and maintaining 96% cell viability at the CQDs content used for device fabrication. The TENG was further demonstrated as a self-powered smart ring for Morse-code-based information transmission.

Overall, these studies demonstrate that nanofillers can serve as functional modules beyond mechanical reinforcement and conductivity enhancement. Multifunctional nanofiller design thereby broadens the role of H-TENGs from energy harvesters to integrated soft platforms for sensing, health care, communication, and wearable electronics.

## 4 Multiscale Design Principles

The preceding sections discussed how nanofillers improve the mechanical, electrical, interfacial, and functional properties of hydrogel-based triboelectric nanogenerators (H-TENGs). However, practical device performance is not determined by filler properties alone. Output stability, mechanical reliability, and wearable applicability emerge from coupled design factors across multiple length scales. Therefore, nanocomposite hydrogel-based TENGs should be understood as multiscale soft energy systems, in which nanoscale filler–matrix interactions, microscale hydrogel networks, mesoscale interfaces, and macroscale device operation are jointly optimized (Table 2). This perspective links material-level strategies to device-level performance and practical use.

**Table 2:** Multiscale Design Principles for Nanocomposite Hydrogel-Based TENGs

design scale	major target	key strategies	main trade-off
nanoscale	- dispersion stability	- filler selection	- filler aggregation
	- local reinforcement	- optimal loading	- increased stiffness
	- charge transport	- surface modification	- electrical pathway disruption
		- interfacial compatibilization	
microscale	- network toughness	- double networks	- conductivity–stretchability balance
	- pathway continuity	- dynamic cross-linking	- toughness–hysteresis balance
	- recovery	- aligned fillers	
		- multicomponent networks	
mesoscale	- charge generation	- structured tribolayers	- contact enhancement–durability balance
	- contact stability	- piezoelectric hydrogel	- protection–sensitivity balance
	- interfacial coupling	- elastomer encapsulation	
macroscale	- wearable conformity	- form-factor design	- conformability–stability balance
	- deformation transfer	- attachment control	- output–system complexity balance
	- usable power	- power-management design	

### 4.1 Nanoscale Filler–Matrix Interactions

At the nanoscale, the key design objective is to control filler–matrix interactions rather than simply increase filler loading. Each filler class has distinct surface chemistry, dimensionality, and electrical characteristics. As summarized in Table 1, nanofillers contribute to H-TENGs mainly by reinforcing hydrogel networks, improving charge transport, or modulating interfacial charge behavior. Therefore, filler selection should be guided by the target function of the device rather than by conductivity or mechanical reinforcement alone.

A major nanoscale challenge is filler aggregation. Excessive filler loading can cause aggregation or restacking, leading to local stress concentration and disrupted transport pathways.^52^ These effects often reduce stretchability and destabilize electrical output under repeated deformation. Thus, the optimal filler content is not necessarily the highest loading level, but the range where dispersion, interfacial bonding, and mechanical compliance are balanced. Surface modification and interfacial compatibilization are useful because they improve polymer–filler coupling and suppress aggregation. Therefore, nanoscale design should aim to stabilize local mechanical and electrical interactions without sacrificing softness and stretchability.

### 4.2 Microscale Hydrogel Network Architecture

At the microscale, local filler–matrix interactions should be organized into continuous hydrogel network structures. The performance of H-TENGs depends not only on the filler type, but also on how fillers are distributed within the polymer network. A well-designed microscale architecture should support stress dissipation, charge transport, and structural recovery during repeated deformation.

Double-network^48,53,57^ and dynamically cross-linked hydrogels^46,47,50^ provide useful strategies for improving mechanical robustness. In these systems, rigid or reversible networks dissipate mechanical energy while soft polymer networks maintain elasticity and recoverability. This network-level design is important for H-TENGs because deformation-induced damage can disrupt charge-transport pathways and gradually reduce the triboelectric output.

The transport architecture is another key factor. Randomly dispersed fillers often require high loading to form conductive pathways, which can compromise stretchability. In contrast, aligned filler arrangements^58^ or multicomponent filler networks^56^ can improve the pathway continuity within the hydrogel matrix. This approach is useful for balancing conductivity, toughness, and deformability.

### 4.3 Mesoscale Triboelectric Interface and Device Assembly

At the mesoscale, the properties of nanocomposite hydrogels must be converted into stable device structures. In many H-TENGs, the hydrogel serves as a soft electrode and is encapsulated by an elastomeric tribolayer. Therefore, the hydrogel–elastomer interface, contact geometry, and encapsulation structure strongly affect charge generation and output stability. Even a highly conductive and stretchable hydrogel may show poor device performance if interfacial adhesion or contact stability is insufficient.

The triboelectric interface remains a major bottleneck. Limited surface charge density restricts electrical output, while repeated contact–separation cycles can cause surface wear and interfacial mismatch. Interface-level output enhancement has been demonstrated mainly through structural surface engineering^44,60^ of the triboelectric layer, such as roughened Ecoflex surfaces and microstructured PDMS charge-generating layers. These structures can increase effective contact area, improve contact conformity, and enhance charge separation at the triboelectric interface. Piezoelectric nanocomposite hydrogel electrodes^65,74^ can further strengthen deformation-induced polarization when coupled with surface-engineered charge-generating layers. These strategies should be used to improve charge generation without compromising contact durability.

Encapsulation is also an important mesoscale design factor because it defines the hydrogel–elastomer interface and the mechanical coupling between the soft electrode and triboelectric layer. In many H-TENGs, elastomer encapsulation is used to isolate the hydrogel from the external environment and reduce water loss, while maintaining stretchability and skin compatibility. However, the encapsulation layer should not be considered only as a passive barrier. Its layer geometry and interfacial coupling can influence contact deformation, signal sensitivity, and long-term output stability.^41,42^ Therefore, elastomer selection and interfacial bonding should be designed together with the triboelectric contact geometry.

### 4.4 Macroscale Wearable Operation and System Integration

At the macroscale, nanocomposite hydrogel-based TENGs should be configured as wearable device structures that conform to the geometry and motion of the target wearing site. Intrinsic material properties, such as stretchability and conductivity, do not automatically lead to practical device performance unless stable mechanical coupling is maintained during operation. Therefore, macroscale geometric factors, including active area, device thickness, and attachment configuration, should be tailored to the deformation mode of the intended wearing site.^27^ For example, bending-type devices require efficient strain transfer along curved joints, whereas pressure- or tactile-sensing configurations require conformal contact and repeatable normal contact–separation. In this sense, macroscale design focuses on converting body-scale motion into stable deformation at the triboelectric interface.

Beyond mechanical conformity, macroscopic integration should also consider the electrical pathway from the H-TENG to external devices. The output characteristics of a TENG are affected by device geometry, contact frequency, and external load, and the generated signal is typically alternating, intermittent, high voltage, and low current.^76^ Therefore, energy-harvesting applications require appropriate rectification, load matching, and energy-storage components to convert the generated output into usable device-level power.^77^ Thus, macroscale system integration should connect wearable form factors, mechanical boundary conditions, and power-management configurations, rather than treating the nanocomposite hydrogel as an isolated material or standalone energy harvester.

In summary, nanocomposite hydrogel-based triboelectric nanogenerators (TENGs) have emerged as promising soft energy devices by integrating the mechanical compliance, tunable ionic conductivity, and skin conformability of hydrogels with the reinforcing, conductive, charge-modulating, and multifunctional roles of nanofillers. In this Mini-Review, recent progress in nanofiller-engineered hydrogel-based TENGs was discussed from the perspectives of mechanical reinforcement, electrical pathway engineering, nanofiller dispersion control, output modulation, and multifunctionality. Representative nanofillers, including nanoclays, MOFs, carbon-based fillers, MXenes, piezoelectric particles, and quantum dots, can strengthen hydrogel networks, construct charge-transport pathways, regulate interfacial polarization, and introduce additional functions. These advances show that nanofillers are not merely passive additives, but active design components that determine the structure–property relationships of hydrogel-based TENGs.

Despite substantial progress, the practical implementation of nanocomposite H-TENGs remains limited by several coupled challenges, including filler aggregation, unstable conductive or ionic pathways, mechanical fatigue, dehydration or freezing, interfacial mismatch, limited output power, and insufficient device-level reliability (Figure 5). Addressing these issues requires moving beyond simple filler addition toward integrated control over filler organization, hydrogel network structure, triboelectric interface stability, environmental adaptability, and system-level power management. In particular, scalable processing, robust encapsulation, stable contact geometry, and standardized long-term testing will be essential for translating material-level demonstrations to reliable wearable devices.

**Figure 5:**
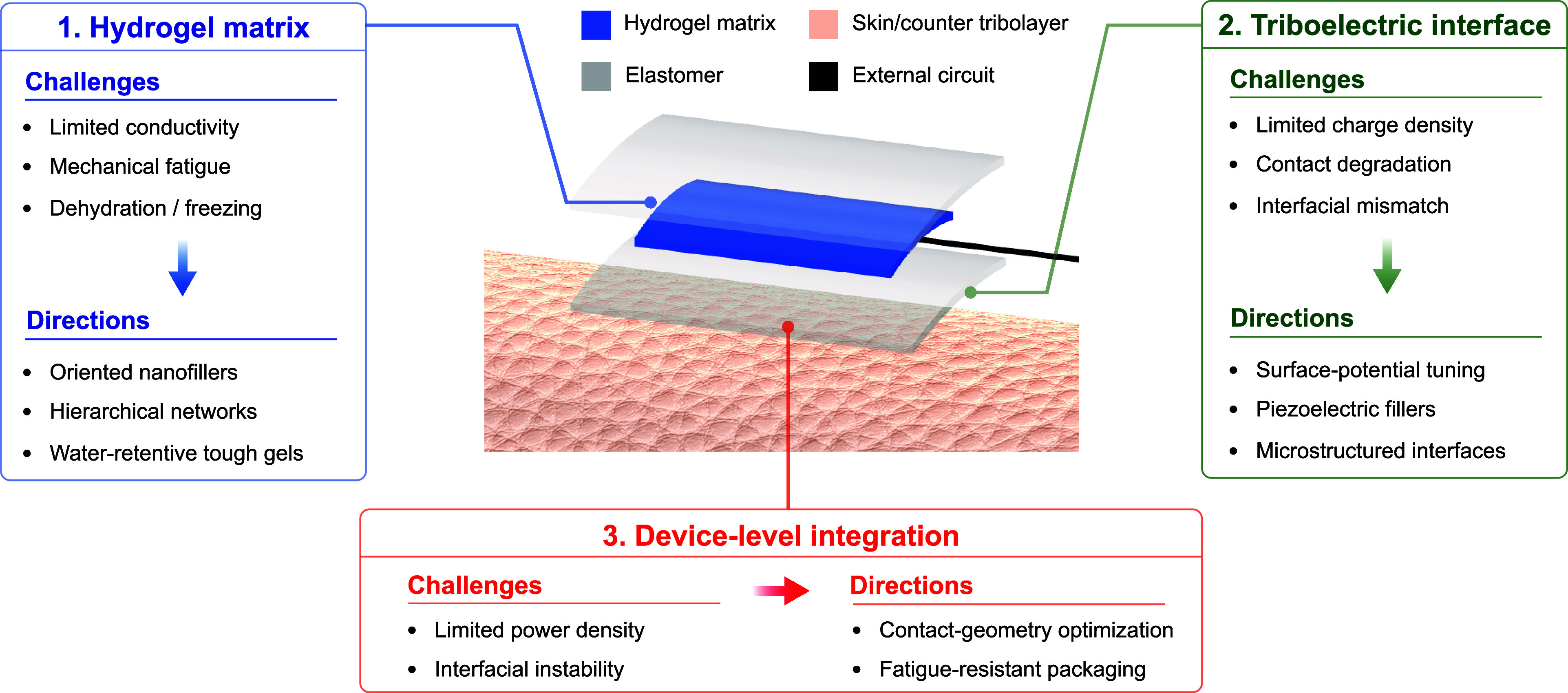
**Outlook for practical implementation of nanocomposite hydrogel-based TENGs.** Future progress requires coordinated advances in hydrogel matrix engineering, triboelectric interface engineering, and device-level integration to address limited conductivity, mechanical fatigue, dehydration/freezing, limited charge density, contact degradation, interfacial mismatch, limited power density, and interfacial instability.

Future progress in nanocomposite hydrogel-based TENGs will depend on integrated multiscale design that connects nanofiller–matrix interactions, hydrogel network mechanics, interfacial charge regulation, and device-level reliability. Future progress should move beyond simple filler addition toward coordinated control over filler organization, charge generation, environmental stability, and system integration. With advances in scalable processing, robust packaging, power management, and device-level integration, these materials could evolve from laboratory-scale soft energy harvesters and self-powered sensors into practical wearable platforms for healthcare monitoring, human–machine interfaces, intelligent wearables, and distributed soft electronics.

## Supplementary Material



## Notes

Comparison of representative nanocomposite hydrogel-based TENGs categorized by the nanofiller design strategy, including hydrogel/nanofiller compositions, selected nonoutput parameters related to mechanical properties, electrical conductivity, durability, sensing performance, and antibacterial activity, and reported open-circuit voltage and short-circuit current values (PDF)

## Abbreviations

TENG: triboelectric nanogenerator
H-TENG: hydrogel-based triboelectric nanogenerator
AI: artificial intelligence
3D: three-dimensional
AC: alternating current
VOC: open-circuit voltage
ISC: short-circuit current
EDL: electric double layer
SVP: streaming vibration potential
MOF: metal–organic framework
ZIF-8: zeolitic imidazolate framework-8
BTO: barium titanate
GO: graphene oxide
GNP: graphene nanoplatelet
CNTs: carbon nanotubes
HO-mCNT: hydroxylated multiwalled carbon nanotubes
CQDs: carbon quantum dots
CNF: cellulose nanofiber
TEMPO: 2,2,6,6-tetramethylpiperidin-1-oxyl
TOCNF: TEMPO-oxidized cellulose nanofiber
PVA: poly­(vinyl alcohol)
PEGDA: poly­(ethylene glycol) diacrylate
PEG: poly­(ethylene glycol)
PEO: poly­(ethylene oxide)
OEG: oligo­(ethylene glycol)
OEGMA: oligo­(ethylene glycol) methyl ether methacrylate
DEGMA: di­(ethylene glycol) methyl ether methacrylate
PAM/PAm/PAAm: polyacrylamide
PAA: poly­(acrylic acid)
PVP: poly­(vinylpyrrolidone)
PEDOT:PSS: poly­(3,4-ethylenedioxythiophene):poly­(styrenesulfonate)
PSS: poly­(4-styrenesulfonic acid)
PVDF-HFP: poly­(vinylidene fluoride-*co*-hexafluoropropylene)
PPy: polypyrrole
SA: sodium alginate
TA: tannic acid
PDA: polydopamine
LM: liquid metal
DL: demethylated lignin
DN: double network
ICH: ion-conducting hydrogel
NIR: near-infrared
EHD: electrohydrodynamic
CPTMS: 3-chloropropyltrimethoxysilane
FOTS: 1*H*,1*H*,2*H*,2*H*-perfluorooctyltriethoxysilane
UFMPH: UiO-66­(Zr)-F4MOF-reinforced piezoelectric hydrogel
PMP DN ICH: poly­(ionic liquid)/MXene/PVA double-network ion-conducting hydrogel
