# Supplementary material for: Multiscale Design Principles for Nanocomposite Hydrogel-Based Triboelectric Nanogenerators
Source: Nano Lett. 2026 Jul 24;26(30):9699–715. doi: 10.1021/acs.nanolett.6c02504 (PMC13450453; doi:10.1021/acs.nanolett.6c02504)
Supplement: Supplementary file 1 [file nl6c02504_si_001.pdf]

Supplementary Material

Multiscale Design Principles for Nanocomposite Hydrogel-Based Triboelectric Nanogenerators

Authors: James Sangmin Choo[1‡], Jinsoo Na[1‡], Siheon Lee[1‡], and Juhyuk Park[1,2,3,4,]*

Affiliations:

[1] Department of Materials Science and Engineering, Seoul National University, Seoul 08826, Republic of Korea

[2] Research Institute of Advanced Materials (RIAM), Seoul National University, Seoul 08826, Republic of Korea

[3] Interdisciplinary Program in Bioengineering, Seoul National University, Seoul 08826, Republic of Korea

[4] SOFT Foundry Institute, Seoul National University, Seoul 08826, Republic of Korea

*Corresponding author. E-mail: juhyukp@snu.ac.kr

Table S1. Representative nanocomposite hydrogel-based TENGs categorized by nanofiller design strategy.

Category	Hydrogel/nanofiller	Key non-output parameter	V_{oc}	I_{sc}	Ref.
Mechanical reinforcement	PEGDA / Laponite	strain 1001.8%	10.4 V	–	[37]
	OEGMA-DEGMA / Laponite	stretchability ~450%	13 V	9 nA	[38]
	PAm/PVP / ZIF-8	elongation 944.2%; hysteresis <7%	85.0 V	–	[40]
Electrical pathway engineering	PVA / HO-mCNT	conductivity 1.94 mS m^{-1}	398 V	4.60 μA	[46]
	PVA/SA / GO	–	~495 V	~22 μA	[49]
	PVA / MXene	stretchability 1800%	230 V	270 nA	[52]
	PAM/SA / MXene	Gauge factor 6.6	150 V	2 μA	[54]
Nanofiller dispersion control	PAM / TOCNF/TA@MXene	stretchability >800%	106 V	~2 μA	[59]
	PVA/PEO / GNP-PSS	endurance >16,000 cycles	594 V	40 μA	[63]
	PAM / PDA@CNTs	self-healing 97.3%; response 76 ms	–	–	[73]
	PAA / DL@LM	toughness 3.77 MJ m^{-3}	272 V	–	[71]
Triboelectric output modulation	PAM / $BaTiO_3$	sensitivity 7.91 V N^{-1}	52.92 V	0.95 μA	[65]
	PAM/CNF / MXene	Gauge factor 6.73	67.5 V	–	[58]
	PVA/PEG / CPTMS- or FOTS-MXene	–	190/212 V	–	[64]
	PVA/PPy / UiO-66(Zr)-F_4	tensile stress 6.88 MPa	–	–	[60]
Multifunctionality	3D-printed PVA / SnS_2/SnS	–	29.0/37.5 V	1.23/1.58 μA	[69]
	PVA / PDA-CNTs	self-healing efficiency 86% after 10 min	38.57 V	7.98 μA	[62]
	Poly(ionic liquid)/PVA / MXene	antibacterial 99.91/99.98%	66.0 V	0.18 μA	[75]
	agar-PAAm / CQDs	inhibition zone 26.8 mm; cell viability 96%	120 V	5.7 μA	[70]

Note: A dash (–) indicates that the value was not reported or not specified in the corresponding reference. The reported electrical outputs were measured using different device configurations and testing conditions, including device geometry, active area, contact material, contact force, deformation mode, operating frequency, external load, and measurement setup. Therefore, the values should be interpreted as representative performance indicators rather than directly comparable metrics across studies.
